# The emerging role of fibrocytes in ocular disorders

**DOI:** 10.1186/s13287-018-0835-z

**Published:** 2018-04-13

**Authors:** Feng Zhang, Ke Liu, Han Zhao, Yan He

**Affiliations:** 1 Department of Ophthalmology, The Second Xiangya Hospital, Central South University, Changsha, 410011 Hunan Province China; 2 Hunan Clinical Research Center of Ophthalmic Disease, Changsha, 410011 Hunan Province China

**Keywords:** Fibrocytes, Mesenchymal progenitor cell, Fibrosis, Inflammation, Ocular disorders

## Abstract

The fibrocyte, which was first described in 1994, is a type of circulating mesenchymal progenitor cell in the peripheral blood. Fibrocytes play important roles in chronic inflammation, wound healing, tissue remodeling, and fibrosis. Emerging evidence indicates that fibrocytes are involved in a wide variety of ocular disorders associated with inflammation and fibrosis. In this review, we summarize recent advances regarding the general characteristic profile of fibrocytes, molecular mechanisms underlying the fibrocyte recruitment to target tissues, their differentiation into fibroblasts, and the potential role of fibrocytes in ocular disease. Given the critical role of fibrocytes in ocular disorders, fibrocytes may serve as a promising pharmaceutical target in the development of novel therapeutic strategies to treat ocular inflammation and fibrosis.

## Background

Adult bone marrow contains a large number of distinct stem or progenitor cells, including hematopoietic stem cells, mesenchymal stem cells, endothelial progenitor cells, and fibrocytes. It has been suggested that other progenitor cells, as well as hematopoietic stem cells, could be involved in the process of hematopoiesis support, neovascularization, and tissue regeneration or wound healing, supporting the blood cells [1–3]. Fibrocytes are among these other progenitor cells, with the features of both lymphocytes and fibroblasts, that have become increasingly researched recently. Circulating fibrocytes were first identified in 1994 in an in vivo study using an animal model of wound repair and were defined by their unique co-expression of hematopoietic and progenitor cell markers (CD45 and CD34, respectively), together with the production of extracellular matrix (ECM) [4]. In addition, fibrocytes express a number of chemokine receptors and adhesion molecules, some of which are critical for the recruitment of fibrocytes to sites of tissue injury, fibrosis, and inflammation [5–7].

Due to the properties of mesenchymal stem cells, fibrocytes are capable of differentiating into several cell lineages, such as classic trilineage cells (including adipocytes, osteoblasts, and chondrocytes), fibroblasts, and myofibroblasts [8, 9]. The function of these cells has been shown to be involved in several pathological processes encompassing fibrosis, inflammation, neovascularization, and some immunological diseases. A large number of in vitro and in vivo studies have revealed distinct roles of fibrocytes in a wide variety of ocular disorders. In this review, we will focus on the latest advances regarding the emerging roles of fibrocytes in ocular disease and the therapeutic potential of specific treatments targeting fibrocytes.

## Origin and phenotypic characteristics

The term fibrocyte, which combines the term fibroblast with leukocyte, thrombocyte, and erythrocyte, was coined for the peripheral blood circulating fibroblast progenitor that produces collagen and also expresses the hematopoietic marker CD34 [10]. Since there are various studies that have focused on fibrocytes involved in diverse pathogeneses, including fibrosis and inflammation, different views of the origin of fibrocytes have arisen. In 1994, Bucala and his colleagues first described a subpopulation of spindle-shaped adherent cells that expressed collagen, CD34, and CD45. They named these cells “fibrocytes”, which represented about 10% of the whole cell population in the wound chamber [4].

Fibrocytes constitute 0.1–0.5% of circulating non-erythrocytes and were isolated from the peripheral blood monocytes [4, 11]. Thus, historically, it was believed that fibrocytes were one type of monocyte in the peripheral blood. However, emerging evidence indicates that fibrocytes are more likely to be originally derived from monocytes [12–14]. Their presumed monocyte origin is proved by the expression of CD11b and CD11c. Interestingly, monocytes consisting of a mixed population of progenitors likely replenish the tissue-resident macrophage and dendritic cell populations in the absence of inflammation after an initial differentiation into different subtypes of monocytes before they enter the tissues. During inflammatory processes, however, a population of monocytes directly migrates to inflamed sites, predominantly through a CCR2-mediated signaling pathway, and differentiate into fibrocytes as a player in inflammation and tissue repair [15, 16]. There are several distinct regulators involved in the process of fibrocytes differentiation. An animal study revealed that differentiation of fibrocytes is critically dependent on CD4^+^ T cells and T-cell activation, which determines whether the development of fibrocytes is supported or blocked [17]. Cytokines produced by Th1 and Th2 cells, respectively (such as interleukin (IL)-4, IL-13, IL-2, and tumor necrosis factor (TNF)), inhibit or promote fibrocyte fate [18, 19]. In serum-free media, some peripheral blood monocytes differentiated into fibrocytes within 5 days, and such differentiation was inhibited by the blood plasma protein serum amyloid P (SAP) [13] likely through its interaction with FcγRI [20]. Other factors affecting fibrocyte differentiation include hyaluronic acids and CD44. High molecular weight hyaluronic acid (HWMHA) potentiates the differentiation of human monocytes to fibrocytes and, in contrast, low molecular weight hyaluronic acid (LWMHA) inhibits fibrocyte differentiation. CD44 may be involved in the regulation of the process of fibrocyte differentiation, with a dominance hierarchy of SAP > LMWHA > HMWHA > IL-4 or IL-13 [21]. The number of circulating fibrocytes was not decreased when a monoclonal antibody against CCR2 was used to deplete monocytes in a mouse model, suggesting that fibrocytes develop outside the kidney independent of infiltrating monocytes and rely on CCR2 for migration into target organs.

**Fig. 1:**
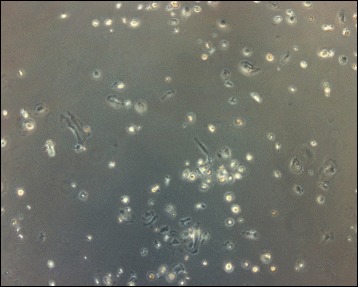
The morphology of cultured fibrocytes under microscopy; 5× magnification

**Fig. 2:**
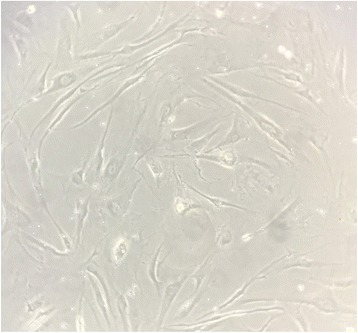
The morphology of cultured fibroblasts under microscopy; 10× magnification

Circulating fibrocytes, a slender spindle-shaped cell type (Fig. 1), exhibit several phenotypic characteristics attributed to the variety of their properties. Consistent with their bone marrow or hematopoietic origin, fibrocytes express CD45, leukocyte-specific protein-1 (LSP-1), and CD34 (a hematopoietic progenitor marker) [22]. Although fibrocytes produce collagen or ECM they were not discovered until the early 1990s. This is likely due to an underestimation of fibrocyte counts as they progressively lose CD34 and CD45 [23]. Moreover, collagen could be a noisy marker to discriminate between other lineages because of its overlap expression by fibroblasts or macrophages [24]. To address this confusion, Pilling et al. screened fibrocytes, monocytes, and macrophages with commercially available reagents to identify markers that could accurately discriminate between these populations, together with their different morphology [24]. One of their principal findings was that only fibrocytes (50–200 μm long spindle-shaped cells with an oval nucleus) expressed CD45RO, 25F9, and S100A8/A9, but not PM-2 K, which could distinguish fibrocytes from other populations [24]. Some additional characteristics and markers of fibrocytes are summarized in Table 1. As mentioned above, there is currently no unified or specific standard for identifying fibrocytes. Fibrocytes have the characteristics of both macrophages and fibroblasts. To differentiate fibrocytes from macrophages and fibroblasts, positive staining of intracellular collagen, fibronectin and vimentin, the co-expression of specific markers (such as CD34, CD45, CXCR4), and the presence of at least several unique features indicated in Table 1 would be an appropriate criterion for defining fibrocytes. In addition, fibroblasts constitute the main resident cells of connective tissue and are considered in a state of activation. In fact, active fibroblast morphology is different from fibrocytes. Fibroblasts have a branched cytoplasm surrounding an elliptical, speckled nucleus (Fig. 2), and, in contrast, inactive fibrocytes are small and spindle-shaped.

**Table 1:** Properties and markers used to identify fibrocytes

Property	Expression
Functions	
Cytokine production	++
Immune cell trafficking	++
ECM production	++
α-SMA production	+
Lipid metabolism	+
Antigen presentation	++
Angiogenesis	+
MMP production	+
Chitinase production	+
Growth factors (TGF-β1, MCP-1)	+/++
Monocyte markers	
CD11a, CD11b, CD11c, CD13, CD32, CD64	+/++
CD14	+/−
CD16	–
Stem cell/progenitor markers	
CD34	++
CD105	++
Dendritic cell markers	
CD1a, Cd10, CD83	–
B cell markers	
CD19	–
T cell markers	
CD3, CD4, CD8, CD25, CD56	–
Macrophages markers	
CD45RO, 25F9, S100A8/A9	+
PM-2 K	–
Integrins	
CD18, CD29, CD49b, CD49e, CD61	++
CD49a	+
CD49c, CD49d, CD49f, CD103, α_4_β_7_	–
Cell surface enzymes	
CD10, CD172a	+
proly14-hydroxylase	+
FAP	+
Scavenging receptors and molecules involved in host defense	
CD68, CD163, CD206, CD209, CD35, CD36	+/−
Chemokine receptors	
CCR1, CCR2, CCR3, CCR4, CCR5, CCR7, CCR9, CXCR1, CXCR4, CX3CR1, CXCR3	+/++
Antigen presentation	
CD40, CD54, CD80, CD86, MHC class I and II	++/+
Extracellular matrix proteins	
Collagen I, III and IV, vimentin, tenascin	+
Fibronectin, α-SMA	+/−
Collagen V	++
MMP-9	++
Glycosaminoglycans	
Perlecan, versican, hyaluronan	++/+
Decorin, biglycan	+
Adhesion and motility markers	
CD43, CD164, galectin 3, LSP1	+/++
CD29, CD44, CD81, ICAM1, CD81	+
Miscellaneous	
Semaphorin 7A	+
CD115	–
CD90	+/−
CD105	+
CD70	–
vWF	–

The symbols represent high or increasing level of expression (++, +), conflicting reports or equivocal evidence of expression level (+/−), and no expression (−), and have been arbitrarily assigned to each marker based on published data [6, 7, 22]

*α-SMA* α-smooth muscle actin, *CCR* CC-chemokine receptor, *CXCR* CXC-chemokine receptor, *ECM* extracellular matrix, *FAP* fibroblast activation protein, *ICAM1* intercellular adhesion molecule 1, *LSP1* leukocyte-specific protein-1, *MCP-1* monocyte chemotactic protein 1, *MMP* matrix metalloproteinase, *TGF-β1* transforming growth factor-β1, *vWF* Von Willebrand factor

## Proliferating and transforming cytokines of fibrocytes

Several studies have identified a profibrotic lung phenotype in aging mice characterized by an increase in the number of fibroblasts lacking the expression of thymocyte differentiation antigen 1 (Thy-1) and an increase in transforming growth factor (TGF)-β1 expression [23, 25, 26]. These findings suggest that TGF-β1 epigenetically regulates the lung fibroblast phenotype through methylation of the Thy-1 promoter [24, 27, 28]. Targeted inhibition of DNMT in the right clinical context might prevent fibroblasts from myofibroblast transdifferentiation and collagen deposition, which in turn could prevent fibrogenesis in the lung and other organs [21, 29]. However, the proliferation ability of fibrocytes in vitro is limited; they cannot proliferate indefinitely because of the Hayflick limit.

## Homing chemokines of fibrocytes (CXCL12/CXCR4)

Fibrocytes can migrate to the site of injury and inflammation, mediated by various chemokine receptors (Table 1), and are able to produce numerous cytokines which take part in the process of inflammation or tissue remodeling. Once there, they differentiate into myofibrocytes and begin the process of tissue remodeling which ultimately culminates in fibrosis [6]. The pivotal CXCL12/CXCR4 axis in fibrocyte chemotaxis, which is implicated in the recruitment process of fibrocytes, has recently attracted attention [26, 30]. Both anti-CXCL12 antibody and direct CXCR4 antagonism have shown protective effects against the development of fibrosis by blocking the fibrocyte trafficking [27, 28].

Inhibition of the mammalian target of rapamycin (mTOR) pathway, a possible upstream factor of CXCL12/CXCR4, with rapamycin has been shown to effectively reduce the recruitment of fibrocytes into tracheal allografts and mitigates the development of tracheal luminal fibrosis. Some other signaling pathways such as CCL2/CCR2 and soluble factors including TNF, IL-10, monocyte chemotactic protein 1 (MCP-1), IL-1, and IL-33 were shown, at least in part, to relate to the migration of fibrocytes in distinct fibrotic diseases [31–33]. Following the recruitment to the tissues, fibrocytes were thought to differentiate into myofibroblasts, driven by TGF-β1, IL-4, and IL-13, and exhibit upregulation of α-smooth muscle actin (α-SMA) and the progressive loss of CD34 and CD45 expression [34].

## Function

It is complicated to elucidate all the functions of fibrocytes involved in various pathologic processes; however, we might conclude that fibrocytes play different roles to some extent, mainly in inflammation and fibrosis. In other words, there are a number of distinct cytokines produced by fibrocytes that play their corresponding roles during the different stages of disorders. Early studies have shown that fibrocytes express α-SMA and are able to contract collagen gels in vitro, revealing their potential to differentiate into myofibroblasts and contribute to wound contraction [8]. Fibrocytes also produce soluble mediators that induce myofibroblast transformation in culture such as platelet-derived growth factor (PDGF) and TGF-β1 [35], and have been shown to control angiogenesis via secretion of soluble mediators including growth factors (TGF-β, PDGF-A, and fibroblast growth factor (FGF)-7), chemokines (MCP-1 and macrophage inflammatory protein (MIP)-1α), and ECM (collagen I and α-SMA) [36]. As one of the most potent fibrogenic factors, TGF-β1 may facilitate fibroblast transformation both in vivo and in vitro in various fibrotic diseases [25, 37–39]. Wang et al. showed that fibrocytes from humans with chronic airway obstruction could transform to myofibroblasts induced by TGF-β1 in vitro [38]. Neveu et al. considered that TGF-β1 epigenetically regulated the lung fibroblast phenotype in vivo, and inhibition of TGF-β1 DNA methyltransferase could prevent fibrogenesis in the lung and other organs [25]. In the injured liver, a sharp release of TGF-β1 was observed to accompany liver fibrosis, and helped in triggering fibrocyte recruitment to the liver injury site and promoting their differentiation [37, 39]. In fibrotic kidney diseases, several clinical trials and experimental models used pharmacological blockade of TGF-β1 as an antifibrotic therapy to improve or slow the decline in kidney function [40–42]. Moreover, in response to IL-1β, fibrocytes were induced by the secretion of IL-6, IL-8, CCL2, CCL3, and intercellular adhesion molecule-1 (ICAM-1) which would be expected to recruit inflammatory cells [11].

## Fibrocyte involvement in ocular disorders

Given the conjunction of ongoing inflammation and fibrosis present in many ocular complications, fibrocytes have been proposed to be a critical player in ocular disease. To date, accumulated evidence has demonstrated the involvement of fibrocytes in a wide variety of ocular disorders, including thyroid-associated orbitopathy (TAO), age-related degeneration (AMD), degenerative retinal diseases, intraretinal revascularization, subfoveal choroidal neovascularization (CNV), postoperation scar formation following trabeculectomy, corneal endothelial dystrophy, pterygial fibrous tissues, and vitreomacular traction syndrome and macular hole. A better understanding of cellular mechanisms underlying the regulation of fibrocytes in the initiation and progression of ocular disorders will shed light on the identification of novel therapeutic strategies to treat ocular disease.

## Thyroid-associated orbitopathy

**Fig. 3:**
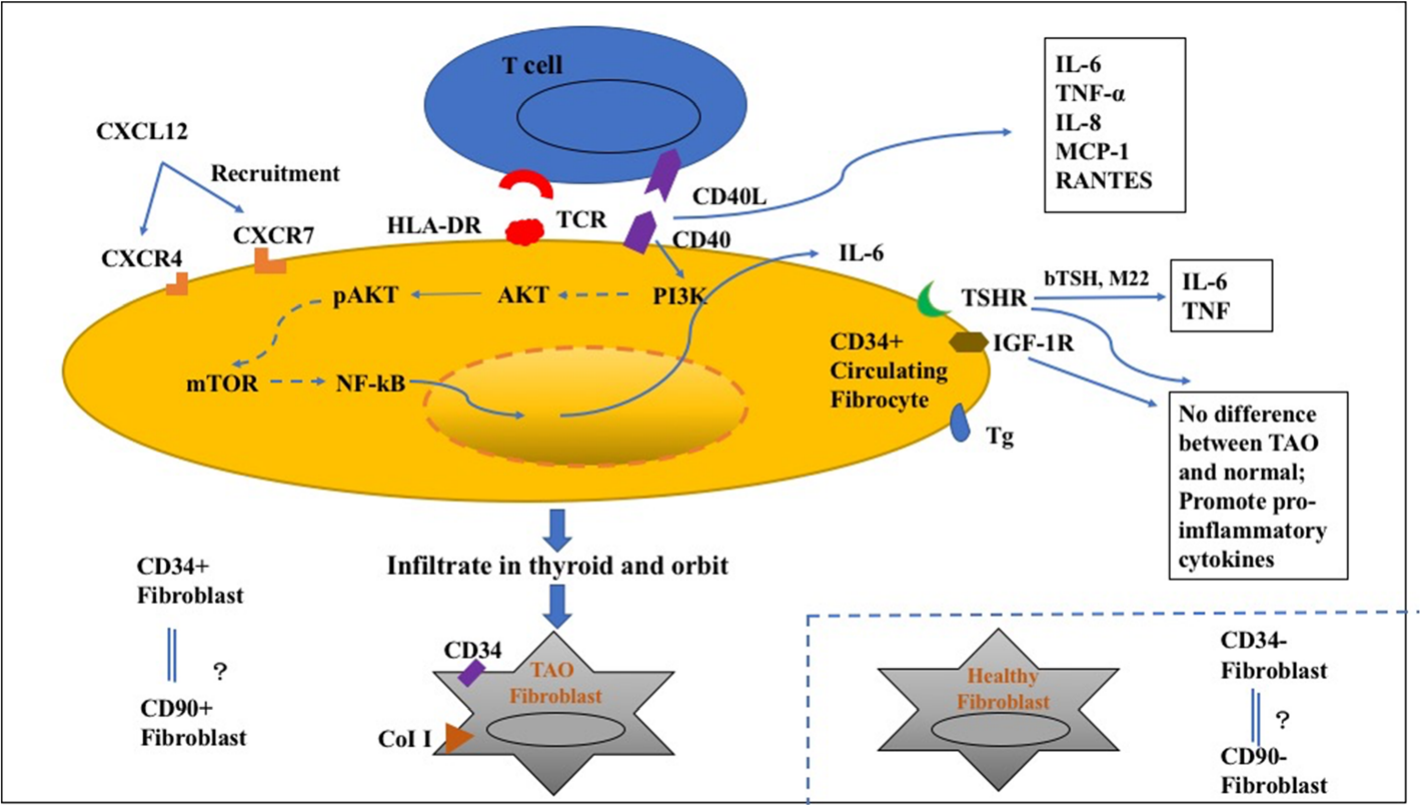
A model of fibrocytes in the pathogenesis of thyroid-associated orbitopathy (TAO). Fibroblasts are uniformly CD34^−^ in healthy humans and CD34^+^ in TAO patients. Fibrocytes appear to infiltrate the orbit in TAO and transition into CD34^+^ orbital fibroblasts, which express much higher levels of thyroglobulin (Tg) and thyroid-stimulating hormone receptor (TSHR), and mediate orbital inflammation and remodeling. Bovine thyroid-stimulating hormone (bTSH) and M22 are TSHR inhibitors. *HLA-DR* human leukocyte antigen D-related, *IGF-1R* insulin-like growth factor-1 receptor, *IL* interleukin, *MCP-1* monocyte chemotactic protein 1, *mTOR* mammalian target of rapamycin, *TCR* T-cell receptor, *TNF* tumor necrosis factor

TAO is an immune-mediated inflammatory disorder usually associated with Grave’s disease (GD) that causes enlargement of the orbital muscles and fat. The molecular mechanisms of TAO are poorly understood. In the past, orbital fibroblasts were thought to be the principal cell type that could give rise to the exophthalmos of patients with TAO, whether or not they were predominantly of the fat or muscle type. Fibrocytes have been shown to be related to inflammation and fibrosis in diverse pathogenesis of tissue remodeling-related disease [22, 43]. Douglas and colleagues found that peripheral blood mononuclear cells (PBMCs) isolated from GD patients yielded approximately fivefold more fibrocytes compared with the control healthy population; however, fibrocyte yields were not statistically different in active TAO patients compared with those with stable disease, and the severity of exophthalmos of the more affected orbit failed to correlate with fibrocyte yields [44]. Thyroid-stimulating hormone receptor (TSHR) is an essential antigen of GD expressed at high levels on fibrocytes isolated from patients, consistent with the study by Gillespie et al. [45] that circulating fibrocytes expressed markedly increased TSHR and proinflammatory chemokines in response to TSH. What was more surprising was that the expression of TSHR on fibrocytes was comparable with that found on cultured thyrocytes. In contrast, undifferentiated orbital fibroblasts, even those from patients with GD, failed to express detectable TSHR. These findings revealed that fibrocytes might be involved in the inflammatory response by activating TSHR and producing various chemokines [45]. In healthy humans fibroblasts are uniformly CD34^−^, and of great potential importance was the finding that those expressing TSHR fibroblasts were uniformly CD34^+^, strongly suggesting that they derive from circulating fibrocytes [46–48]. In addition, fibrocytes defined as CD34^+^ and LSP-1^+^ infiltrated to orbital tissues in large amounts, suggesting that they may migrate to the orbit and mediate tissue reactivity and remodeling through local production of cytokines such as IL-6 and TNF-α [47]. The same group then carried out an in-depth study where they suggested that fibrocytes displayed particularly high levels of functional CD40. CD40/CD40 ligand binding raised the production of several proinflammatory cytokines, amongst which IL-6 expression was mediated through the Akt and NF-κB pathways [49]. The expression and function of CD40 on fibrocytes also suggested that they might provide the antigen-specific T-cell reaction [49]. Douglas’ team also showed that CD40 expression in fibrocytes is induced by TSH and mediates IL-8 expression [50, 51]. Fernando et al. reported that fibrocytes from GD patients not only express TSHR, but also express thyroglobulin (Tg); moreover, GD orbital fibroblasts, which contain CD34^+^ and CD34^−^ cells, express much lower levels of Tg and TSHR [47]. All these findings indicated that fibrocytes from GD patients are characteristic of multiple thyroid-specific markers by which it potentially shares the derivation with the fibroblast in GD patients. Reducing signaling from TSHR on fibrocytes could be a useful strategy in treating TAO. A theoretical schematic of the roles of fibrocytes in TAO is shown in Fig. 3.

## Vitreoretinopathy

Despite many risk factors associated with vitreoretinopathy, inflammation and fibrosis are thought to be the predominant cause in the pathologic process of different ocular fibroproliferative diseases. Proliferative diabetic retinopathy (PDR), one of the proliferative vitreoretinopathies and sequelae of diabetes mellitus, is caused by the inflammation and metabolic changes resulting from diabetes mellitus, which results in vascular leakage with alteration of the phenotype of fibrocytes [52]. Fibrocytes were detected in the vitreous and fibrovascular membrane in PDR patients. Cultured cells from the vitreous of PDR patients exhibited a spindle shape and expressed fibrocyte markers, and these cells could differentiate into myofibroblasts via TGF-β1. These findings indicate that fibrocytes might be involved in the development of PDR [53]. An earlier study revealed that circulating fibrocytes migrated to the epiretinal membranes of proliferative vitreoretinopathy (PVR), differentiated into myofibroblasts, and contributed to wound healing predominantly via the chemotactic CXCL12/CXCR4 pathway [54].

Moreover, an ultrastructural study of patients with vitreomacular traction syndrome revealed that myofibroblasts are the predominant cell type in the epiretinal tissue and the inner limiting membrane of 12 out of 14 specimens [55]. Other ocular fibrotic processes, such as macular hole, also showed that fibrocytes were in the tissue removed at the time of surgery [29]. Taken together, these finding suggested that circulating fibrocytes are a precursor of myofibroblasts in pathologic epiretinal membranes, consistent with the effects of fibrocytes in the process of inflammation and fibrosis of other systemic diseases.

## Corneal wound healing

The stromal opacity during corneal wound healing is a process associated with precipitation of ECM and myofibroblast generation and persistence. Animal studies revealed that PRM-151, a recombinant form of human pentraxin-2 (also referred to as serum amyloid P), modulated the generation of myofibroblasts after opacity-producing corneal injury in rabbits by inhibiting differentiation of circulating monocytes into fibrocytes and profibrotic macrophages [56]. This finding indicates that PRM-151 inhibited myofibroblast generation after opacity induction, and also suggests that fibrocytes contribute to corneal myofibroblast generation. Furthermore, other investigators have found that bone marrow-derived cells could differentiate into myofibroblasts, with increased expression of α-SMA in the corneal stroma after irregular phototherapeutic keratectomy, and that the presence of these cells within the cornea is associated with corneal stromal haze.

## Pterygium fibrosis

Pterygium is commonly observed in ocular pathological fibrosis and consists of elastotic degeneration of collagen and fibrovascular degeneration [57]. Strong immunoreactivities against progenitor cell markers such as CD34, c-kit, vascular endothelial growth factor (VEGF) receptor 1 and 2 were detected in surgically removed pterygium tissue [58]. CXCR4-positive cells aggregated in the pterygium stroma in response to stromal cell-derived factor (SDF)-1 and might represent the process of differentiation for monocyte precursors into myofibroblasts [59]. A probable cellular mechanism of pterygium formation is that dysregulated healing signaling exceeds the requirement for the recovery of tissue damage, and thereby limbal basal cells will be changed to abnormally altered pterygial cells, and the excessive wound healing process and remnant, altered cells resulted in recurrence using the same mechanism. Clinically, patients with pterygium treated by temporary amniotic membrane patch after pterygium removal were found to have circulating CD34^+^ cells, which represent the active subset of fibrocytes, increased slightly compared with a marked increase in the bare sclera group; this might represent an effective therapeutic approach for controlling pain—lower collagen expression, and excessive infiltration of bone marrow-derived stem cells [60].

## Other ocular disorders

Given that they might be a pivotal player in inflammation and fibrosis, circulating fibrocytes have drawn increased attention for the study of ocular disease. Two representative examples of such ocular disorders are postoperation scar formation following trabeculectomy and choroidal neovascularization.

## Conclusions and future directions

A growing body of literature over the last decade has shown that circulating fibrocytes may serve as an important source of fibroblasts and myofibroblasts during normal or aberrant reparative processes in diverse fibrotic disorders associated with inflammation. Fibrocytes have been implicated in many inflammatory and fibrotic ocular diseases, and their presence at high levels is one of the biomarkers for TAO. Previous studies have demonstrated that both growth factor-induced and hypoxia-driven CXCR4 expression is mediated through the PI3K/mTOR pathway and can be inhibited by rapamycin, which substantially diminished the accumulation of fibrocytes in target tissues. Therefore, the PI3K/AKT/mTOR/CXCR4 signaling pathway may serve as a promising pharmaceutical target to treat ocular disorders. The molecular mechanisms underlying the transformation and differentiation process of fibrocytes into fibroblasts and myofibroblasts remains largely unclear, which also reminds us that fibrosis is not exclusively caused by fibroblasts. How these relative cells correlate with each other phenotypically, how they lose expression of some critical markers (e.g., CD34, CD90), and the inhibition of the pathway should be the concern for the future for a possible therapeutic strategy.

In summary, fibrocytes and the fibrotic process may serve as novel targets for intervention in chronic inflammatory eye diseases. A better understanding of the identity and characteristics of fibrocyte subsets as well as their regulatory mechanisms in ocular disorders will provide valuable insights into the identification of new therapeutic strategies.

## Abbreviations

AMD: Age-related degeneration
CNV: Choroidal neovascularization
ECM: Extracellular matrix
GD: Grave’s disease
IL: Interleukin
LSP-1: Leukocyte-specific protein-1
MCP-1: Monocyte chemotactic protein 1
mTOR: Mammalian target of rapamycin
PBMC: Peripheral blood mononuclear cell
PDGF: Platelet-derived growth factor
PDR: Proliferative diabetic retinopathy
PVR: Proliferative vitreoretinopathy
SAP: Serum amyloid P
SMA: Smooth muscle actin
TAO: Thyroid-associated orbitopathy
Tg: Thyroglobulin
TGF: Transforming growth factor
Thy-1: Thymocyte differentiation antigen 1
TNF: Tumor necrosis factor
TSHR: Thyroid-stimulating hormone receptor

## References

[1] Pontikoglou C (2011). Bone marrow mesenchymal stem cells: biological properties and their role in hematopoiesis and hematopoietic stem cell transplantation. Stem Cell Rev..

[2] Takahashi T (1999). Ischemia- and cytokine-induced mobilization of bone marrow-derived endothelial progenitor cells for neovascularization. Nat Med..

[3] Wynn TA (2008). Cellular and molecular mechanisms of fibrosis. J Pathol..

[4] Bucala R (1994). Circulating fibrocytes define a new leukocyte subpopulation that mediates tissue repair. Mol Med..

[5] Rankin SM (2012). Chemokines and adult bone marrow stem cells. Immunol Lett..

[6] Bellini A, Mattoli S (2007). The role of the fibrocyte, a bone marrow-derived mesenchymal progenitor, in reactive and reparative fibroses. Lab Investig..

[7] Reilkoff RA, Bucala R, Herzog EL (2011). Fibrocytes: emerging effector cells in chronic inflammation. Nat Rev Immunol..

[8] Abe R (2001). Peripheral blood fibrocytes: differentiation pathway and migration to wound sites. J Immunol..

[9] Mori L (2005). Fibrocytes contribute to the myofibroblast population in wounded skin and originate from the bone marrow. Exp Cell Res..

[10] Bucala R (2008). Circulating fibrocytes: cellular basis for NSF. J Am Coll Radiol..

[11] Chesney J (1998). Regulated production of type I collagen and inflammatory cytokines by peripheral blood fibrocytes. J Immunol..

[12] Cox N, Pilling D, Gomer RH (2012). NaCl potentiates human fibrocyte differentiation. PLoS One..

[13] Pilling D, Gomer RH (2012). Differentiation of circulating monocytes into fibroblast-like cells. Methods Mol Biol..

[14] Curran TA, Ghahary A (2013). Evidence of a role for fibrocyte and keratinocyte-like cells in the formation of hypertrophic scars. J Burn Care Res.

[15] Gordon S, Taylor PR (2005). Monocyte and macrophage heterogeneity. Nat Rev Immunol..

[16] Tacke F, Randolph GJ (2006). Migratory fate and differentiation of blood monocyte subsets. Immunobiology..

[17] Niedermeier M (2009). CD4+ T cells control the differentiation of Gr1+ monocytes into fibrocytes. Proc Natl Acad Sci U S A..

[18] Shao DD (2008). Pivotal advance: Th-1 cytokines inhibit, and Th-2 cytokines promote fibrocyte differentiation. J Leukoc Biol..

[19] Medina A, Ghahary A (2011). Reprogrammed fibrocytes induce a mixed Th1/Th2 cytokine response of naive CD4(+) T cells. Mol Cell Biochem..

[20] Crawford JR, Pilling D, Gomer RH (2012). FcgammaRI mediates serum amyloid P inhibition of fibrocyte differentiation. J Leukoc Biol..

[21] Maharjan AS, Pilling D, Gomer RH (2011). High and low molecular weight hyaluronic acid differentially regulate human fibrocyte differentiation. PLoS One..

[22] Peng H, Herzog EL (2012). Fibrocytes: emerging effector cells in chronic inflammation. Curr Opin Pharmacol..

[23] Keeley EC, Mehrad B, Strieter RM (2011). The role of fibrocytes in fibrotic diseases of the lungs and heart. Fibrogenesis Tissue Repair..

[24] Pilling D (2009). Identification of markers that distinguish monocyte-derived fibrocytes from monocytes, macrophages, and fibroblasts. PLoS One..

[25] Neveu WA (2015). TGF-beta1 epigenetically modifies Thy-1 expression in primary lung fibroblasts. Am J Physiol Cell Physiol..

[26] Garibaldi BT (2013). Regulatory T cells reduce acute lung injury fibroproliferation by decreasing fibrocyte recruitment. Am J Respir Cell Mol Biol..

[27] Harris DA (2013). Inhibiting CXCL12 blocks fibrocyte migration and differentiation and attenuates bronchiolitis obliterans in a murine heterotopic tracheal transplant model. J Thorac Cardiovasc Surg..

[28] Song JS (2010). Inhibitory effect of CXC chemokine receptor 4 antagonist AMD3100 on bleomycin induced murine pulmonary fibrosis. Exp Mol Med..

[29] Green WR (2006). The macular hole: histopathologic studies. Arch Ophthalmol..

[30] Gillen JR (2013). Rapamycin blocks fibrocyte migration and attenuates bronchiolitis obliterans in a murine model. Ann Thorac Surg..

[31] Bianchetti L (2012). IL-33 promotes the migration and proliferation of circulating fibrocytes from patients with allergen-exacerbated asthma. Biochem Biophys Res Commun..

[32] Mathai SK (2010). Circulating monocytes from systemic sclerosis patients with interstitial lung disease show an enhanced profibrotic phenotype. Lab Investig..

[33] Hara A, et al. CCL2/CCR2 augments the production of transforming growth factor-beta1, type 1 collagen and CCL2 by human CD45−/collagen 1-positive cells under high glucose concentrations. Clin Exp Nephrol. 2013;17(6):793–804. 10.1007/s10157-013-0796-6 23564379

[34] Hong KM (2007). Differentiation of human circulating fibrocytes as mediated by transforming growth factor-beta and peroxisome proliferator-activated receptor gamma. J Biol Chem..

[35] Hartlapp I (2001). Fibrocytes induce an angiogenic phenotype in cultured endothelial cells and promote angiogenesis in vivo. FASEB J.

[36] Kao HK (2011). Peripheral blood fibrocytes: enhancement of wound healing by cell proliferation, re-epithelialization, contraction, and angiogenesis. Ann Surg..

[37] Xu J, Kisseleva T (2015). Bone marrow-derived fibrocytes contribute to liver fibrosis. Exp Biol Med (Maywood)..

[38] Wang CH (2008). Increased circulating fibrocytes in asthma with chronic airflow obstruction. Am J Respir Crit Care Med..

[39] Kisseleva T, Brenner DA (2008). Fibrogenesis of parenchymal organs. Proc Am Thorac Soc..

[40] Boon MR (2011). Bone morphogenetic protein 7: a broad-spectrum growth factor with multiple target therapeutic potency. Cytokine Growth Factor Rev..

[41] Lee SY, Kim SI, Choi ME (2015). Therapeutic targets for treating fibrotic kidney diseases. Transl Res..

[42] Qin W (2011). TGF-beta/Smad3 signaling promotes renal fibrosis by inhibiting miR-29. J Am Soc Nephrol..

[43] Blakaj A, Bucala R (2012). Fibrocytes in health and disease. Fibrogenesis Tissue Repair.

[44] Douglas RS (2010). Increased generation of fibrocytes in thyroid-associated ophthalmopathy. J Clin Endocrinol Metab..

[45] Gillespie EF (2012). Increased expression of TSH receptor by fibrocytes in thyroid-associated ophthalmopathy leads to chemokine production. J Clin Endocrinol Metab..

[46] Li B, Smith TJ (2013). Divergent expression of IL-1 receptor antagonists in CD34(+) fibrocytes and orbital fibroblasts in thyroid-associated ophthalmopathy: contribution of fibrocytes to orbital inflammation. J Clin Endocrinol Metab..

[47] Fernando R (2012). Human fibrocytes coexpress thyroglobulin and thyrotropin receptor. Proc Natl Acad Sci U S A..

[48] Schmidt M (2003). Identification of circulating fibrocytes as precursors of bronchial myofibroblasts in asthma. J Immunol..

[49] Gillespie EF (2012). Interleukin-6 production in CD40-engaged fibrocytes in thyroid-associated ophthalmopathy: involvement of Akt and NF-kappaB. Invest Ophthalmol Vis Sci..

[50] Douglas RS (2014). Thyrotropin receptor and CD40 mediate interleukin-8 expression in fibrocytes: implications for thyroid-associated ophthalmopathy (an American Ophthalmological Society thesis). Trans Am Ophthalmol Soc..

[51] Mester T (2016). CD40 Expression in fibrocytes is induced by TSH: potential synergistic immune activation. PLoS One..

[52] Shiels IA (1998). Vascular leakage stimulates phenotype alteration in ocular cells, contributing to the pathology of proliferative vitreoretinopathy. Med Hypotheses..

[53] Tamaki K (2016). Fibrocytes and fibrovascular membrane formation in proliferative diabetic retinopathy. Invest Ophthalmol Vis Sci..

[54] Abu El-Asrar AM (2008). Circulating fibrocytes contribute to the myofibroblast population in proliferative vitreoretinopathy epiretinal membranes. Br J Ophthalmol..

[55] Gandorfer A, Rohleder M, Kampik A. Epiretinal pathology of vitreomacular traction syndrome. Br J Ophthalmol. 2002;86(8):902–9. 10.1136/bjo.86.8.902 PMC1771255 12140213

[56] Santhiago MR (2011). Monocyte development inhibitor PRM-151 decreases corneal myofibroblast generation in rabbits. Exp Eye Res..

[57] Safi H (2016). Correlations between histopathologic changes and clinical features in pterygia. J Ophthalmic Vis Res..

[58] Kim KW (2013). Upregulated stromal cell-derived factor 1 (SDF-1) expression and its interaction with CXCR4 contribute to the pathogenesis of severe pterygia. Invest Ophthalmol Vis Sci..

[59] Kim KW, Park SH, Kim JC (2016). Fibroblast biology in pterygia. Exp Eye Res..

[60] Lee JK (2010). The change of cytokines in tear and blood after different pterygium operation. Cytokine..

